# Effects of Ginseng Supplementation and Endurance-Exercise in the Artery-Specific Vascular Responsiveness of Diabetic and Sedentary Rats

**DOI:** 10.3389/fphys.2018.00460

**Published:** 2018-05-04

**Authors:** Juan M. Murias, Mao Jiang, Tomasz Dzialoszynski, Earl G. Noble

**Affiliations:** ^1^Faculty of Kinesiology, University of Calgary, Calgary, AB, Canada; ^2^School of Kinesiology, University of Western Ontario, London, ON, Canada

**Keywords:** endothelium-dependent vasorelaxation, vessel myography, type I diabetes, vascular kinetics, aerobic training

## Abstract

This study examined the effects of 12 weeks North-American ginseng supplementation, exercise training, and sedentary behavior on vascular responses in type I diabetic rats. The following hypotheses were tested: (1) ginseng supplementation would result in improved vascular responsiveness and sensitivity; (2) exercise training would result in further improvement in these vascular responses; (3) control rats with no access to exercise would show a depressed vascular response compared to control rats that were not exposed to a sedentary lifestyle. Groups: non-diabetic sedentary control (C_S_), sedentary diabetic (D_S_), sedentary diabetic with ginseng supplementation (D_S_+GS), diabetic with ginseng supplementation and high-intensity endurance exercise (D+GS+EX), and control not exposed to sedentary behavior (C). Diabetes was induced by streptozotocin. Arteries were excised, cleaned, and mounted onto a myography system. Percent vasorelaxation to acetylcholine (ACh) (10^-8^ M ACh to 10^-4^ M ACh) of the carotid artery was similar in C_S_ (57 ± 31%), C (66 ± 35%), D_S_ (58 ± 36%), D+GS+Ex (71 ± 37%), and D_S_+GS (64 ± 37%) (*p* > 0.05). Percent vasorelaxation of the aorta was smaller in C_S_ (23 ± 17%) compared to C (46 ± 35%), D_S_ (60 ± 40%), D+GS+Ex (64 ± 40%), and D_S_+GS (56 ± 39%) (*p* < 0.05), and smaller in C compared to D+GS+Ex (*p* < 0.05). In the femoral, the percent vasorelaxation was reduced in D_S_ (18 ± 16%) compared to all the other conditions (C_S_, 43 ± 22%; C, 79 ± 28%; D+GS+Ex, 55 ± 27%; D_S_+GS, 45 ± 26%; *p* < 0.05), but larger in C compared to the other conditions (C_S_, D_S_, D+GS+Ex, D_S_+GS; *p* < 0.05). Diabetes and sedentary lifestyle have detrimental effects on vascular responses that are evident in the femoral arteries of the diabetic rats. Ginseng supplementation restored the loss of sensitivity, with no added vascular protection of exercise training.

## Introduction

Provision of O_2_ and nutrients to different organs and tissues through appropriate distribution of blood flow is of critical importance for cellular homeostasis (Behnke and Delp, 2010). Several studies have shown that endothelium-dependent mechanisms related to release of nitric oxide (NO) through activation of endothelial NO synthase (e-NOS) are important for diffusion of NO to the smooth muscles, and thus to the amplitude (i.e., dose–response) (Furchgott and Zawadzki, 1980; Stamler et al., 1994; Dimmeler et al., 1999) and the kinetics components (Murias et al., 2013a,b) of vascular relaxation. Type I diabetes mellitus has been shown to have detrimental effects on vascular responsiveness through different mechanisms so that NO availability is reduced and vasorelaxation responses are impaired (Johnstone et al., 1993; Hadi and Suwaidi, 2007; Murias et al., 2013b). More specifically, increases in oxidative stress and reactive oxygen species (ROS) (Ting et al., 1996; Beckman et al., 2003) as well as inflammation (MacKenzie, 2011), conditions commonly observed in diabetes, have been linked to this reduced responsiveness in the vasculature.

Different interventions have proven to be beneficial in restoring diabetes-related loss of vascular responsiveness and sensitivity. For instance, as an alternative to the use of prescribed drugs, non-pharmaceutical treatments such as exercise training programs have shown positive effects on the vasculature (Fuchsjager-Mayrl et al., 2002; Goto et al., 2003; Murias et al., 2013b). However, exercise training is not always a viable alternative in diabetic patients and indeed it may increase the risk of hypoglycemia (Bhatia and Wolfsdorf, 1991). As such, other natural options for restoring or improving vascular function in diabetic populations should be investigated. In this regard, although the use of ginseng (one of the most popular herbal medicine) has been shown to improve vascular protection in healthy subjects (Chen, 1996) and in diabetic populations (Amin et al., 2011) by means of its antioxidant, anti-inflammatory and vasodilatory effects, there is still a dearth of information on the effects of ginseng supplementation on the dynamic adjustment and sensitivity of blood vessels in diabetes.

As such, the main goals of this study were to: (1) Determine whether or not 12 weeks of North American ginseng (*Panax quinquefolius*) supplementation would help overcoming some of the detrimental effects of diabetes in the vasculature; (2) examine if an endurance exercise intervention would add vascular protection to that potentially obtained with ginseng supplementation. A secondary goal of the study was to compare a control group that had been housed in similar conditions as the diabetic and experimental groups (sedentary lifestyle as a consequence of limited living space), to a control group that had not been exposed to this sedentary lifestyle. We hypothesized that: (1) ginseng supplementation would result in improved vascular responsiveness and sensitivity; (2) exercise training would result in further improvement in these vascular responses; (3) control rats with a sedentary lifestyle would show a depressed vascular response compared to control rats that were not exposed to a sedentary lifestyle.

## Materials and Methods

### Animal Characteristics

This study was approved by the University of Western Ontario Council on Animal Care and was performed in accordance with the guidelines of the Canadian Council on Animal Care. Fifty-eight male 8 week-old Sprague Dawley rats obtained from Charles River Laboratories were housed in pairs in standard rat cages with a 12:12-h light–dark cycle in a temperature (20 ± 1°C) and relative humidity (50%) controlled environment. Food and water were provided *ad libitum*.

### Experimental Protocol

Rats were randomly assigned to a non-diabetic sedentary control (C_S_; *n* = 12), sedentary diabetic (D_S_; *n* = 14), sedentary diabetic with ginseng supplementation (D_S_+GS; *n* = 12), diabetic with ginseng supplementation and high intensity endurance exercise (D+GS+EX; *n* = 12), and a control group that was not exposed to sedentary behavior (C; *n* = 8). Type I diabetes mellitus was induced by giving 20 mg kg^-1^ of streptozotocin (STZ) via intraperitoneal injection on four consecutive days. This approach was selected because it mimics the development of type I diabetes more closely than a single dose injection of STZ (O’Brien et al., 1996). Diabetes was confirmed when two blood glucose concentrations greater than18 mM L^-1^ were measured on consecutive days. Once diabetes was confirmed, animals from all but the C group were maintained an additional 12 weeks under their respective conditions prior to sacrifice. Animals were housed in pairs in cages, with no access to exercise devices such as rat activity wheels. This created an environment of limited physical activity that differed from the breeding facility which had larger cages with up to 30 animals per cage. Animals in the C group were sacrificed 1 week after arriving at the facility (i.e., these 8-week old animals were not exposed to living in a smaller cage with reduced socialization for 12 weeks). The inclusion of the C group was not an *a priori* decision, and this group was included as the responses evaluated in the C_S_ rats, although greater than those observed in the D_S_ group, were still lower than what was expected for control animals.

### Ginseng Supplementation

A lyophilized aqueous extract of North American ginseng was prepared as described previously (Azike et al., 2011). After confirmation of a diabetic state (>18 mM L^-1^ glucose on two consecutive days) animals in the ginseng treatment groups were provided ginseng supplementation in their drinking water. On a daily basis the lyophilized powder from the aqueous extract of ginseng was mixed into the drinking water such that the animals consumed ∼250 mg kg^-1^ of ginseng per day. Animals were weighed twice weekly and ginseng content in the water bottles was adjusted so as to provide the proposed dose. Given that the animals were housed in pairs to comply with the recommendations from the University of Western Ontario Council on Animal Care, the exact rate of water consumption per kg body mass in each rat was not precisely established. However, pilot data from our laboratory indicated that water consumption of individually housed, ginseng supplemented diabetic animals was quite similar across animals. Thus, we feel confident that the animals received approximately the proposed dose over the course of the 12 week study.

### Exercise Training Protocol

Immediately upon confirmation of diabetes, exercise training on a motor-driven treadmill was initiated for the D+GS+EX group. For the first week of the exercise-training program, rats slowly increased their exercise duration so that by the end of week 1 they were running 1 h per day, 5 days per week up a 2% grade at 21 m min^-1^ running speed. This exercise regime was chosen to represent an exercise intensity of ∼70% of VO_2max_ (Bedford et al., 1979). It is acknowledged that precise estimation of the intensity of exercise is difficult to determine. However, given that these were diabetic rats, and based on a previous study using moderate and high endurance training intensities (Murias et al., 2013b), we believe that our estimation is correct and that the animal exercised in the upper region of the heavy intensity domain, but still below critical intensities of exercise (i.e., unsustainable performance). Continuous running during the aerobic exercise sessions was encouraged by small blasts of compressed air and touching on the hindquarters.

### Vessels Collection

Rats were anesthetized via an intraperitoneal injection of 65 mg kg^-1^ pentobarbital sodium and were sacrificed via heart excision. For the exercised rats, 18 h separated the last bout of exercise from vessel collection. The carotid, aorta, and femoral arteries were rapidly excised and placed into ice-cold modified Krebs–Henseleit buffer (118.1 mM NaCl, 4.7 mM KCl, 1.5 mM CaCl_2_, 1.2 mM KH_2_PO_4_, 1.2 mM MgSO_4_, 11.1 mM D-glucose, 25 mM NaHCO_3_, pH 7.4). The vessels were then carefully cleaned of connective and adipose tissue. A portion of the carotid, abdominal aorta, and femoral artery were then divided into ∼2 mm long rings and, after removal of luminal blood clots, vessel rings were used for *in vitro* isometric tension measurements. These vessels were selected to represent a commonly used artery in this type of preparation that reflects more central delivery of blood (i.e., aorta), and more peripheral conduit arteries that, despite similar morphological characteristics, have been shown to display differential functional profiles (i.e., carotid and femoral) (Murias et al., 2013a,c).

### *In Vitro* Isometric Tension Analysis

Each vessel ring was mounted onto a GlobalTown Microtech EZ-bath system (GobalTown Microtech, Inc., Sarasota, FL, United States) and placed in 5 ml organ baths containing modified Krebs–Henseleit buffer (37°C) that was constantly aerated with 95% O_2_ and 5% CO_2_. Initial ring tension was manually adjusted to ∼2 g in the aorta, ∼1.5 g in the carotid, and ∼1.0 g in the femoral artery. These values were the results of pilot testing in our laboratory to determine optimal baseline tensions for each vessel section, as previously reported (Murias et al., 2013a,b,c). Rings were allowed to equilibrate at these tensions for ∼45 min. Fresh buffer (5 ml) was added to organ baths at the end of the equilibration period. Isometric contractions and relaxations were continuously measured using PowerLab (ML856 26T; ADInstruments, Colorado Springs, CO, United States). Data were recorded using LabChart v7.0 (ADInstruments, Colorado Springs, CO, United States) at a sampling rate of 1000 Hz. The vessels were pre-constricted with 10^-5^ M phenylephrine (PE). When a steady-state level of constriction was observed, vasorelaxation of the vessels to a dose of 10^-8^ M acetylcholine (Ach) was measured. After PE and ACh was washed-out from each organ bath via three buffer changes, vessels were equilibrated again within a ∼30-min period for subsequent vasoconstriction and vasorelaxation. This process was repeated on four occasions for measurement of vasorelaxation responses to cumulatively higher doses of the vasoactive substance: 10^-7^ M ACh, 10^-6^ M ACh, 10^-5^ M ACh, and 10^-4^ M ACh. Following these experiments, vessels were once more pre-constricted and then exposed to a single dose of 10^-5^ M of the NOS inhibitor N^G^-nitro-L-arginine methyl ester (L-NAME). Vasorelaxation responses to 10^-4^ M ACh and to 10^-4^ M sodium nitroprusside (SNP) were assessed in the presence of L-NAME in the organ bath.

### Data Analysis

The on-transient vasorelaxation responses were modeled using this equation:

$$Y_{\left(t\right)}=Y_{Bs\ln}-A\left(1-e^{-(t-TD)/\tau }\right)$$

where Y_(_*_t_*_)_ is the tension (g) at any given time (*t*); Y_Bsln_ is the steady state baseline value of Y before a decrease in the tension as a consequence of the vasorelaxation; A is the amplitude of the decrease in Y below Y_Bsln_; τ (time constant of the response) is the time required to attain 63% of the steady-state amplitude; and TD is the mathematically generated time delay through which the exponential model is projected to intersect Y_Bsln_. Vasorelaxation responses were modeled to ∼2 min because the relaxant effects of ACh are known to be transient in nature due to the chemical instability of endothelium-derived relaxing factors (Hattori et al., 1991). The model parameters were estimated by least-squares non-linear regression (Origin, OriginLab, Corp., Northampton, MA, United States) in which the best fit was defined by minimization of the residual sum of squares and minimal variation of residuals around the Y-axis (Y = 0). The 95% confidence interval (CI_95_) for the estimated time constant was determined after preliminary fit of the data with Y_Bsln_, A, and TD constrained to the best-fit values and the τ allowed to vary. The calculated time delay for the vasorelaxation response (CTD) was estimated using second-by-second data and represented the time, after ACh infusion, at which the signal initiated a systematic decrease from its steady-state constriction value. The time-to-steady-state (TTSS) represented the CTD + 4τ (with 4τ being ∼98% of the total adjustment). The overall response represented by the TTSS indicates the speed of adjustment of the vasorelaxation response from the infusion of the vasoactive substance (i.e., ACh) to the steady-state nadir value and is a functional measure of the dynamic response of vasorelaxation of the vessel. Baseline constriction values were calculated as the mean value in the 30 s prior to a transition. For determination of the sensitivity of each vessel in each condition to cumulative doses of ACh, the percent vasorelaxation response was plotted on the y-axis against the dose of ACh on the x-axis.

For calculation of the percent vasorelaxation responses to each dose of ACh as well as ACh subsequent to exposure to L-NAME, and SNP, PE induced contraction of the vessels was deemed 100% tension, and the relaxation responses were calculated as the percent of a stable change in tension relative to the pre-contraction baseline values.

Non-fasted state serum glucose measurements were obtained from blood collected from the abdominal aorta prior to sacrifice using a One Touch Ultra 2 Blood Glucose Monitoring System (Lifescan Canada, Ltd., Burnaby, BC, Canada) and One Touch test strips (Lifescan Canada; range = 0–600 mg/dl).

### Statistical Analysis

Data are presented as means ± standard deviation. For analysis of the kinetics of the vasorelaxation response, a two-way analysis of variance (ANOVA) was used to determine statistical significance for the dependent variables. The ANOVA model was described as G5 × V3 such that groups (C_S_; D_S_; D_S_+GS; D+GS+EX; C) is crossed with vessels (carotid, aorta, and femoral). For the dose–response sensitivity analysis, a two-way repeated measures ANOVA was used to determine statistical significance for the dependent variables. This model was described as G5 × V3 such that groups (C_S_; D_S_; D_S_+GS; D+GS+EX; C) is crossed with vessels (carotid, aorta, and femoral) for each dose (10^-8^ M ACh, 10^-7^ M ACh, 10^-6^ M ACh, 10^-5^ M ACh, 10^-4^ M ACh). A Tukey *post hoc* analysis was used when significant differences were found for the main effects of each dependent variable. The ANOVA was analyzed by SPSS Version 20.0 (SPSS, Inc., Chicago, IL, United States). Dose–response EC_50_ values (the dose that results in 50% of the maximal vasorelaxation to the infusion of ACh) were obtained using dose–response software as described elsewhere (Di Veroli et al., 2015). Statistical significance was declared when *p* < 0.05.

## Results

The C_S_ (617 ± 61 g) and C (257 ± 7 g) rats were significantly heavier and lighter than all other groups, respectively (D_S_, 378 ± 50 g; D_S_+GS, 375 ± 53 g; and D+GS+EX, 408 ± 22 g). No other between group differences were observed for body mass.

The overall vasoconstriction responses were not significantly different between groups (C_S_, 3.2 ± 0.9 g; D_S_, 3.1 ± 0.9 g; D_S_+GS, 2.9 ± 1.0 g; D+GS+EX, 3.0 ± 1.0 g; C, 3.1 ± 1.2 g); *p* > 0.05) with no significant group by vessel interactions observed (*p* > 0.05).

Serum glucose concentrations prior to sacrifice were lower in C_S_ (6.1 ± 1.1 mM L^-1^) and C (4.8 ± 0.3 mM L^-1^) than in D_S_ (20.9 ± 3.4 mM L^-1^), D+GS+Ex (19.4 ± 2.3 mM L^-1^), and D_S_+GS (19.8 ± 3.7 mM L^-1^).

### Kinetics Analysis

**FIGURE 1:**
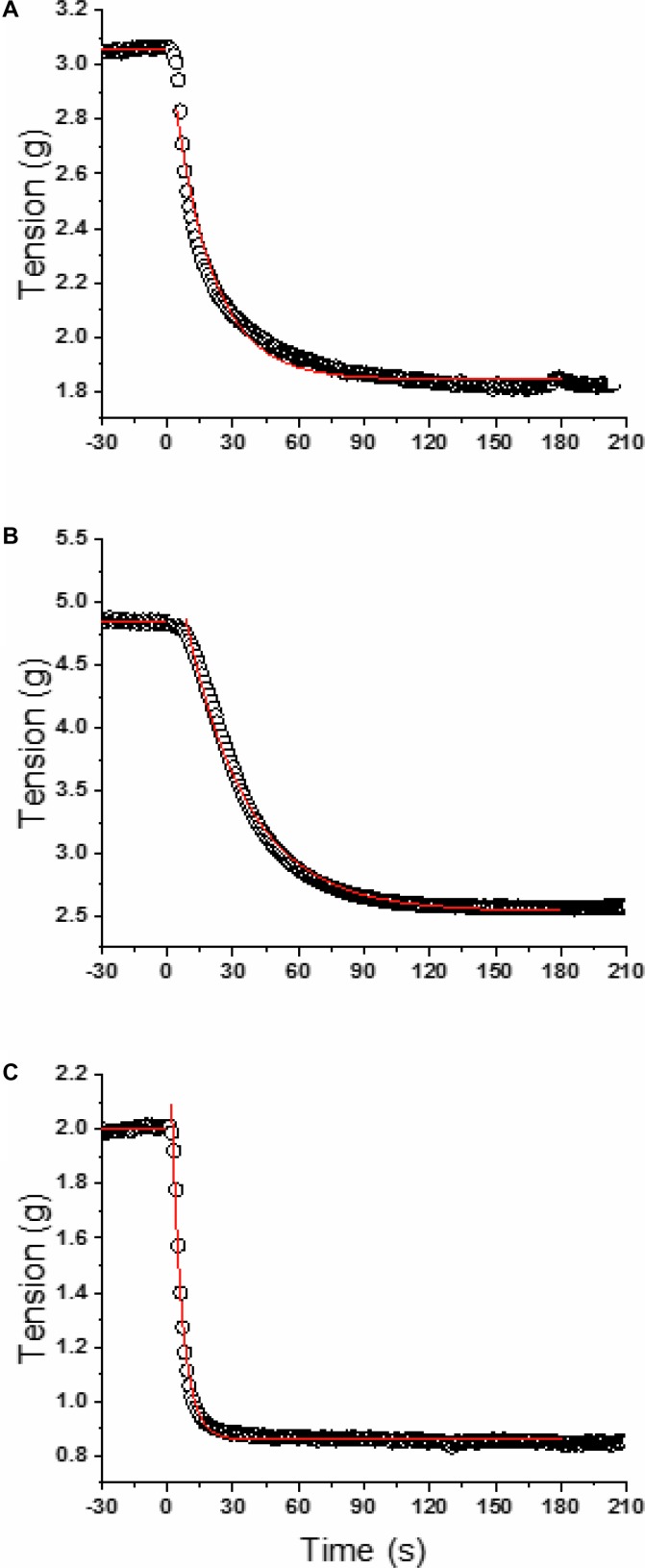
Model fits for a representative carotid **(A)**, aorta **(B)**, and femoral **(C)**. Open circles are the raw data and the red lines represent the linear (baseline) and mono-exponential (ACh infusion starting at time 0) model fits.

**FIGURE 2:**
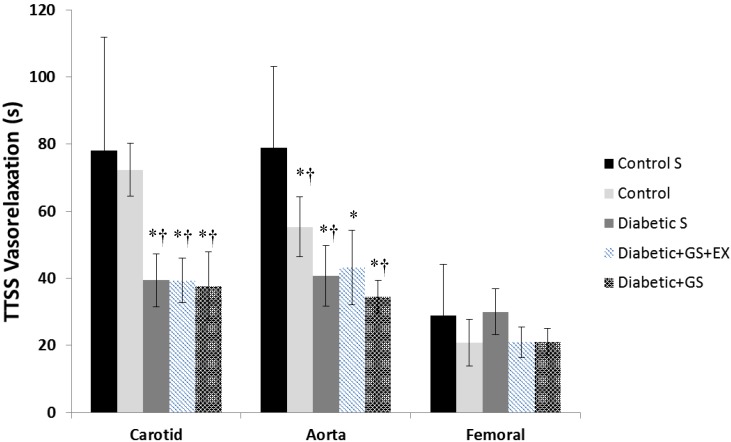
Time-to-Steady-Stated of the vasorelaxation response for each vessel in each experimental conditions. ^∗^ Significantly different from Control Sedentary; † significantly different from Control.

**FIGURE 3:**
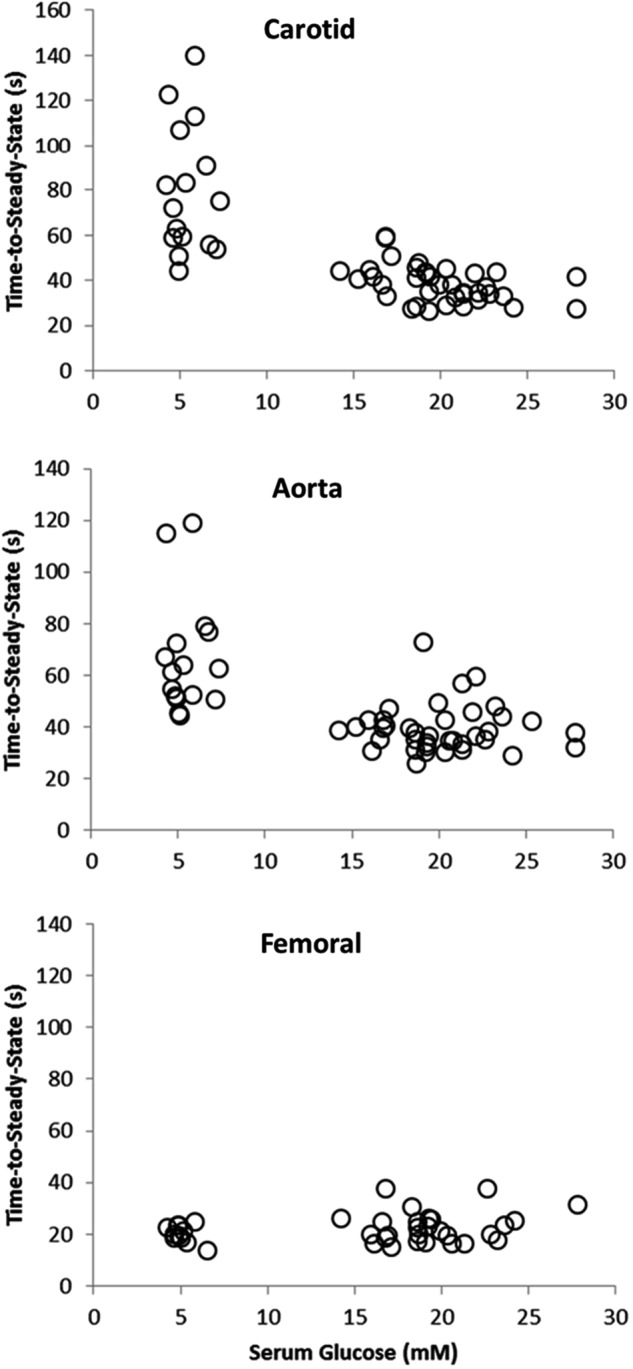
Scatterplot displaying the time-to-steady-state of the vasorelaxation response as a function of serum glucose for the carotid and aorta arteries.

**Figure 1** illustrates a typical vasorelaxation response for a carotid, aorta, and femoral artery with it corresponding kinetics fitting. The dynamic vasorelaxation response for each vessel in each condition as expressed by the TTSS is depicted in **Figure 2**. It should be noted that 50% or less of the femoral vessels showed a vasorelaxation response in C_S_ (6 of 12) and D_S_ (6 of 14). As such, the TTSS is presented for those femoral vessels only. In the carotid, the TTSS was shorter in D_S_ (39 ± 8 s), D+GS+Ex (39 ± 7 s), and D_S_+GS (38 ± 10 s) compared to C_S_ (78 ± 34 s) and C (72 ± 18 s) (*p* < 0.05). In the aorta, TTSS was shorter in C (55 ± 8 s), D_S_ (41 ± 9 s), D+GS+Ex (43 ± 11 s), and D_S_+GS (35 ± 5 s) compared to C_S_ (79 ± 24 s) (*p* < 0.05), and in D_S_ and D_S_+GS compared to C (*p* < 0.05). There was a trend toward a shorter TTSS in D+GS+Ex compared to C (*p* = 0.058). There were no differences in the TTSS for any of the conditions in the femoral artery (C_S_, 29 ± 15 s; C, 21 ± 3 s; D_S_ 30 ± 7 s; D+GS+Ex 21 ± 4 s; D_S_+GS 21 ± 5 s) (*p* > 0.05). **Figure 3** displays the TTSS of the vasorelaxation response as a function of serum glucose for the carotid and aorta arteries.

### Dose–Response Analysis

**FIGURE 4:**
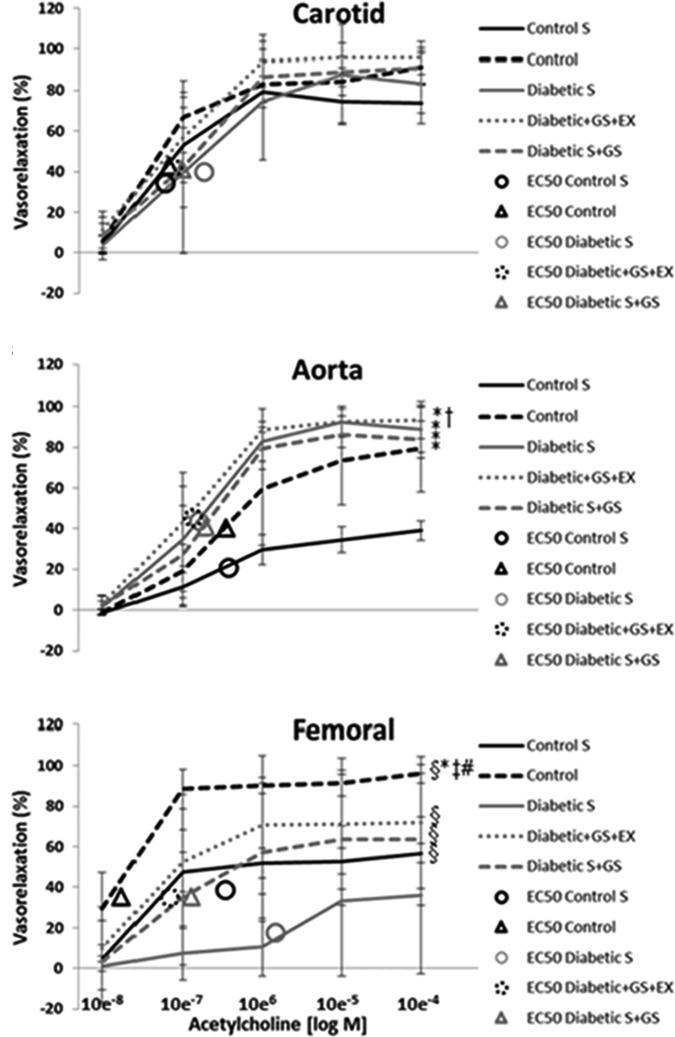
vasorelaxation responses to cumulative doses of ACh. ^∗^ Significantly different from C_S_; † significantly different from C; § significantly different from D_S_; ‡ significantly different from D_S_+GS; # significantly different from D+GS+Ex.

The vasorelaxation responses to cumulative doses of ACh for each vessel in each experimental condition are shown in **Figure 4**.

#### Carotid

The overall % vasorelaxation response (10^-8^ M ACh to 10^-4^ M ACh) of the carotid artery was similar in C_S_ (57 ± 31%), C (66 ± 35%), D_S_ (58 ± 36%), D+GS+Ex (71 ± 37%), and D_S_+GS (64 ± 37%) (*p* > 0.05; **Figure 4A**). The EC_50_ values for C_S_ (5.9 ± 3.4 e^-7^), C (6.6 ± 4.5 e^-7^), D_S_ (1.8 ± 1.3 e^-6^), D+GS+Ex (7.3 ± 4.2 e^-7^), and D_S_+GS (9.6 ± 2.5 e^-7^) were not significantly different (*p* > 0.05).

#### Aorta

The overall % vasorelaxation response in the aorta was smaller in C_S_ (23 ± 17%) compared to C (46 ± 35%), D_S_ (60 ± 40%), D+GS+Ex (64 ± 40%), and D_S_+GS (56 ± 39%) (*p* < 0.05; **Figure 4B**). Additionally, the overall % vasorelaxation response was smaller in C compared to D+GS+Ex. The EC_50_ values for C_S_ (3.7 ± 2.5 e^-6^), C (3.5 ± 4.5 e^-6^), D_S_ (1.6 ± 1.3 e^-6^), D+GS+Ex (1.3 e^-6^ ± 8.2 e^-7^), and D_S_+GS (1.9 ± 1.4 e^-6^) were not significantly different across conditions (*p* > 0.05).

#### Femoral

In the femoral, the overall % vasorelaxation response was reduced in D_S_ (18 ± 16%) compared to all the other conditions (C_S_, 43 ± 22%; C, 79 ± 28%; D+GS+Ex, 55 ± 27%; D_S_+GS, 45 ± 26%) (*p* < 0.05; **Figure 4C**). On the contrary, C displayed a larger % vasorelaxation compared to the other conditions (C_S_, D_S_, D+GS+Ex, D_S_+GS) (*p* < 0.05; **Figure 4C**). The EC_50_ values for D_S_ (1.5 ± 2.3 e^-5^) were significantly greater than those observed in C_S_ (3.4 ± 5.5 e^-6^), C (1.7 e^-7^ ± 9.8 e^-8^), D+GS+Ex (6.7 ± 5.0 e^-7^), and D_S_+GS (1.3 e^-6^ ± 9.6 e^-7^).

### L-NAME and SNP Responses

After being exposed to a single dose of 10^-5^ M of the NOS inhibitor, vasorelaxation to 10^-4^ M ACh was virtually abolished in all conditions and vessels (C_S_: carotid 4 ± 4%, aorta 2 ± 3%, femoral 5 ± 9%; D_S_: carotid 7 ± 4%, aorta 1 ± 2%, femoral 0 ± 1%; D_S_+GS: carotid 5 ± 3%, aorta 1 ± 1%, femoral 1 ± 4%; D+GS+EX: carotid 4 ± 4%, aorta 1 ± 2%, femoral 0 ± 10%; C: carotid 5 ± 5%, aorta 0 ± 2%, femoral 3 ± 12%). With the exception of the overall vasorelaxation response to ACh being greater in the carotid (5 ± 4%) compared to the aorta (1 ± 2%) (*p* < 0.05) but not the femoral (2 ± 12%) (*p* > 0.05), no main effects for condition or vessel by condition interactions were observed (*p* > 0.05). Addition of SNP to the organ bath resulted in virtually full vasorelaxation responses in each condition and vessel (C_S_: carotid 94 ± 9%, aorta 94 ± 9%, femoral 100 ± 7%; D_S_: carotid 96 ± 5%, aorta 96 ± 2%, femoral 102 ± 4%; D_S_+GS: carotid 96 ± 1%, aorta 100 ± 6%, femoral 101 ± 3%; D+GS+EX: carotid 97 ± 3%, aorta 100 ± 3%, femoral 101 ± 7%; C: carotid 92 ± 4%, aorta 95 ± 4%, femoral 98 ± 6%). There were no differences observed between conditions, vessels, or condition by vessel interactions (*p* > 0.05).

Based on the differences observed between the vasorelaxation to 10^-4^ M Ach subsequent to a dose of 10^-5^ M L-NAME, and the vasorelaxation subsequent a dose of 10^-4^ M SNP, the percent of the vasorelaxation that can be mostly attributed to endothelium-dependent mechanisms was as follows: C_S_: carotid 90 ± 11%, aorta 92 ± 8%, femoral 96 ± 15%; D_S_: carotid 90 ± 7%, aorta 95 ± 3%, femoral 103 ± 4%; D_S_+GS: carotid 91 ± 3%, aorta 99 ± 6%, femoral 100 ± 6%; D+GS+EX: carotid 93 ± 8%, aorta 100 ± 3%, femoral 101 ± 15%; C: carotid 87 ± 8%, aorta 97 ± 3, femoral 95 ± 16%.

## Discussion

This study examined the effects of 12 weeks of North American ginseng supplementation and exercise training on vascular responses in diabetic rats. The main findings were that: (1) although the sensitivity and amplitude of the vascular response to ACh of the femoral artery was severely affected by diabetes, this disease did not negatively affect the sensitivity to ACh of the carotid or aorta artery; (2) North American ginseng supplementation restored the loss of sensitivity in the femoral artery, and exercise training did not add any further vascular protection; (3) control sedentary rats showed a depressed percent vascular responsiveness to ACh in the femoral and aorta arteries compared to control animals that were not exposed to 12 weeks of living within a limited space.

In agreement with previous observations from our group (Murias et al., 2013a,b,c), this study demonstrated a vessel-specific vasorelaxation response. The main finding from this investigation was that the sensitivity to ACh of the femoral artery, supplying blood to the locomotor muscles in the hind limbs, was severely affected by diabetes as well as by sedentary behavior, whereas the carotid artery, supplying blood to the brain, showed no detrimental changes in sensitivity from diabetes or from living in an environment that limited movement. This differential response might simply reflect functional characteristics of the vessels that make the femoral artery more likely to be affected by diabetes and lack of activity. This idea is supported by the fact that 6 out of 12 and 6 out of 14 femoral vessels showed a complete abolishment of the vasorelaxation response in the in C_S_ and D_S_ groups, respectively. In these animals, the femoral artery was unable relax in response to cumulative doses of ACh. This lack of endothelium-dependent vasoactive response would profoundly limit the ability of the femoral artery to make adjustments to accommodate blood flow based on increments in metabolic requirements. On the contrary, the carotid artery showed no negative signs related to diabetes or limited physical activity. This is not necessarily surprising as blood supply to the brain is tightly regulated (Vavilala et al., 2002) so that even under the conditions presented in this study, the carotid artery must guarantee similar provision of blood to the brain as that observed in healthy and more active rats. In other words, whereas the stimulus for maintaining endothelium-dependent responses will be fairly constant in the carotid artery independently of diabetes and the limitations for movement imposed by the living environment, the femoral artery will be more affected by the lower metabolic rates associated with the present model. In agreement with this notion, greater endothelium-dependent vasorelaxation and blood flow have been shown in areas of the muscle that are most active due to increased ACh and e-NOS protein expression (Laughlin and Roseguini, 2008). These data do not preclude that longer periods of diabetes or inactivity (i.e., aging) will not affect vasorelaxation responses in the carotid artery as well, but simply reflect that 12 weeks of diabetes or restricted movement were not sufficient to produce detrimental effects in the sensitivity to ACh. Additionally, although conflictive data exists on this issue, it has to be acknowledged that data in humans have demonstrated a link between type I diabetes and carotid artery dysfunction (Vouillarmet et al., 2016) that might be present even in children (Rad et al., 2014). In relation to this, a connection between type I diabetes and cerebrovascular disease and stroke has to be acknowledged and, perhaps, certain lack of translation between animal and human data needs to be considered in this case. Nevertheless, the data from the present study show that, at least in this specific model and testing conditions, functional vasorelaxation responses are vessel-specific.

Importantly, ginseng supplementation resulted in the sensitivity to ACh being re-established in the femoral artery. Although data from the present study cannot determine the mechanisms that mediated this improved functional response, different factors affecting this response could be speculated upon. For instance, it has been shown that *Panax* ginseng helps reverse vascular dysfunction induced by diabetes, and that the protective effects are likely explained by down-regulation of atherosclerosis-related genes and altered lipid metabolism, which contribute to re-establish normal endothelium functions (Chan et al., 2013). Additionally, different experimental studies have established that ginseng extract provide a direct vasodilatory effect on isolated blood vessels (Karmazyn et al., 2011) that may be attributable to endothelium-dependent release of NO (Kwan et al., 2004) secondary to stimulation of NOS activity (Jeon et al., 2000). Indeed, a recent review reported that ginsenosides play a role in stimulating NO production in several systems (Lü et al., 2009). Another mechanism of action for improved vascular response with ginseng supplementation is related to a reduction in ROS, which are more predominant in diabetic populations (Timimi et al., 1998). In this regard, protective effects of ginsenosides have been shown in damaged endothelium from rabbits (Gillis, 1997). Furthermore, other mechanisms such as blockade of calcium channels have also been implicated in the improved vasodilatory response (Kwan et al., 2004). Contrary to our hypothesis, exercise training did not add further improvements to vascular sensitivity compared to ginseng supplementation alone. Previous studies have shown the positive effects of exercise training on vascular responses (Fuchsjager-Mayrl et al., 2002; Goto et al., 2003; Sallam et al., 2010; Murias et al., 2013b) and we speculated that adding exercise training to the ginseng supplementation would result in more pronounced improvements in the vasorelaxation response. In fact, the use of a high intensity of endurance training was determined by a previous study showing that this but not lower intensities of endurance exercise resulted in better vascular adaptations (Murias et al., 2013b). However, in that study, a “controlled” diabetic model was used in which insulin supplementation kept glucose concentrations in the rats in the range of 9–15 mM L^-1^. In the present experimental conditions with animals showing glucose concentrations of ∼20 mM L^-1^, it is likely that some of the beneficial effects that high intensity endurance exercise training exerts on the vasculature were attenuated. For example, this model of type I diabetes with high glucose concentrations would increase oxidative stress (Ting et al., 1996; Timimi et al., 1998; Beckman et al., 2003), which could result in greater production of superoxide and other ROS scavenging NO that would reduce NO bioavailability (Goto et al., 2003).

In addition to the sensitivity (dose–response) of the vessels to ACh, this study examined the dynamic adjustment of each artery (kinetics response) to a dose of 10^-4^ M ACh. Previously, we have demonstrated that diabetes resulted in a slower adjustment of the vasorelaxation response compared to control animals, as shown by a larger τ or TTSS value (Murias et al., 2013a,b). Similarly, it was established that exercise training restored the dynamic adjustment of diabetic rats to what was observed in the control animals (Murias et al., 2013b). Surprisingly, the responses in the present investigation did not agree with previous observations. Indeed, the dynamic adjustment of the vasorelaxation response was faster in all diabetic groups compared to the control groups (both C and C_S_), and this change was mediated by a significant reduction in the TTSS in the carotid and aorta vessels under diabetes. The reasons for this unexpected behavior are unclear, but they might be mediated by the high level of serum glucose concentration. As described in **Figure 2**, when serum glucose concentration in the carotid and the aorta was larger than ∼15 mM L^-1^ (the highest concentration in the previous studies mentioned above), then the TTSS of the response was significantly reduced, and vice-versa. In fact, glucose concentrations lower than 15 mM L^-1^ were associated with a wider range of dynamic adjustments in the carotid and the aorta, as described elsewhere (Murias et al., 2013b,c). Previously, we have interpreted the faster dynamic adjustment in control or in exercise-trained compared to diabetic animals as a positive feature. However, in this case, this faster adjustment, which is even more pronounced in the aorta, is counterintuitive. A previous study has shown enhanced endothelium-dependent vasodilation in the aorta in the early stages of an STZ mouse diabetic model, and attributed this improved response to enhanced production of prostaglandin I_2_ and endothelium-derived hyperpolarizing factor (Shen et al., 2003). Another possibility to consider is the role of the glycocalyx in the observed responses. It has been shown that the glycocalyx, a dynamic layer that is important to vascular homeostasis, is affected by circulating blood glucose concentrations (Van Teeffelen et al., 2007). When circulating glucose concentration values are normal, a thicker glycocalyx might increase the resistance to flow and “slow down” the rate of vasorelaxation while, at the same time, increasing shear stress to produce a larger percent vasorelaxation. A thinner glycocalyx layer as a result of high glucose concentrations would have the opposite results. Nevertheless, although more studies are warranted to mechanistically explain these contradictory responses using different diabetes models, it is important to emphasize that interpretations should be made with caution when trying to extrapolate data from animal models to human responses when the conditions of the model do not closely resemble what is observed in humans (i.e., a rat diabetic model where glucose concentration fluctuates between 9 and 15 mM L^-1^, might better reflect what would be expected in humans compared to a model in which glucose concentrations exceed 20 mM L^-1^).

Another important finding from this study is the depressed vascular responsiveness to ACh observed in the femoral artery of what was defined as the control sedentary compared to the control group. The reason for the selection of the control sedentary group as presented in this investigation was to mimic similar conditions as those presented in the diabetic animals. In that sense, it could be argued that this is the appropriate group against which vasorelaxation responses in the diabetic rats should be compared, as all animals were exposed to identical living conditions. However, it could also be argued that the control sedentary group in this study does not represent the responses that would be observed in healthy animals that were not exposed to a sedentary lifestyle. Under these circumstances, it would remain unclear what part of the detrimental effects in vasorelaxation responses are related to diabetes or to inactive lifestyle *per se*. For instance, if we were to take the control sedentary rats as our reference group, the vasorelaxation response to the highest dose of ACh in the femoral artery of the diabetic sedentary group would have represented ∼63% of that demonstrated in the control sedentary group. However, if we used the control group as our reference, then the vasorelaxation response in the diabetic sedentary group would have represented ∼38% of the total response in relation to this “control” measure. As such, appropriate definition of what a control group represents seems warranted when analyzing physiological responses to a given intervention.

A limitation of this study is that we were unable to collect enough tissue to study some potential mechanisms that might control the changes in functional responses observed in this study. Thus, further research is warranted to elucidate the factors regulating these functional changes. Additionally, it has to be considered that the present results can only be interpreted in relation to the effects of ginseng supplementation on a type I STZ-induced diabetes model. Future studies should examine whether or not ginseng supplementation has similar effect in other groups. Another factor to take into account when interpreting these results is that differences in vascular responsiveness observed between the control and control sedentary rats might also be related to the added stress of living in a cage for 12 weeks in the C_S_ group, and that the model presented in this study has not actually measured physical activity and/or energy expenditure. However, the lack of differences in the carotid makes us think that functional rather than stress related factors might be responsible for the observed differences. Similarly, it has to be acknowledged that the C group was ∼11–12 weeks younger and significantly lighter than the C_S_ group. In relation to the age, although the C groups was younger than the C_S_ group, both were near or within the range of young adult animals (Lewis et al., 2002). As for the differences in body mass, it has to be accepted that the greater mass in the C_S_ group might be associated not only to growth but also to an increase in percent body fat, which has been shown to affect vascular response (Perticone et al., 2001). Regardless, the main point to highlight is that the definition of “control group” needs to be clearly established when making comparisons between groups. Finally, for the present study, length-tension curves were not individually performed for each vessel and the baseline tension for each vessel was based on extensive pilot data examining different baseline tensions for each vessel, as described in the methods. Although we feel confident that the responses are consistently near the largest vasorelaxation for each type of vessel, we cannot rule out that some suboptimal constriction/relaxations might have occurred.

## Conclusion

This study demonstrated that diabetes and sedentary lifestyle have detrimental effects on vascular responses that, at least in the present investigation, are mostly observed in the femoral artery, such that the sensitivity to cumulative doses of ACh is reduced. Importantly, supplementation with herbal medicine ginseng helped in reversing these effects.

## Author Contributions

JM: research design, data collection analysis and interpretation, statistical analysis, manuscript writing and revisions. MJ: research design, data collection and analysis, manuscript revisions. TD: research design, data analysis, manuscript revisions. EN: research design, data interpretation, manuscript revisions.

## Conflict of Interest Statement

The authors declare that the research was conducted in the absence of any commercial or financial relationships that could be construed as a potential conflict of interest.
